# Clinical and molecular cytogenetic findings and pregnancy outcomes of fetuses with isochromosome Y

**DOI:** 10.1186/s13039-022-00611-3

**Published:** 2022-08-04

**Authors:** Yiqun He, Li Guo, Laiping Zheng, Congmian Ren, Ting Wang, Jian Lu

**Affiliations:** Prenatal Diagnosis Centre, Guangdong Women and Children Hospital, 521-523 Xingnan Road, Guangzhou, 511442 Guangdong China

**Keywords:** Isochromosome Y, Idic(Y), I(Y), Mosaicism, Prenatal diagnose, CMA, QF-PCR, FISH

## Abstract

**Background:**

The mosaic forms and clinical phenotypes of fetuses with isochromosome Y are difficult to predict. Therefore, we summarized the cases of nine fetuses with isochromosome Y identified in prenatal diagnosis with a combination of molecular cytogenetic techniques, providing clinical evidence for prenatal genetic counseling.

**Methods:**

The prenatal diagnosis and pregnancy outcomes of nine fetuses with isochromosome Y were obtained by a  retrospective analysis. Isochromosome Y was identified prenatally by different approaches, such as conventional karyotyping, chromosomal microarray analysis (CMA), quantitative fluorescent polymerase chain reaction (QF-PCR) and fluorescence in situ hybridization (FISH).

**Results:**

Seven idic(Y) fetuses and two i(Y) fetuses were identified. One fetus was complete for i(Y)(p10), and the rest with 45,X had mosaic forms. A break and fusion locus was identified in Yp11.3 in one fetus, in Yq11.22 in six fetuses and in Yp10 in two fetuses. The CMA results suggested that different deletions and duplications were found on the Y chromosome. The deletion fragments ranged from 4.7 Mb to the entire Y chromosome, and the duplication fragments ranged from 10.4 to 18.0 Mb. QF-PCR analysis suggested that the AZF region was intact in one fetus, four fetuses had AZFb+c+d deletion, one fetus had AZFa+b+c+d deletion, and one fetus had AZFc+d deletion. Finally, four healthy male neonates were delivered successfully, but the parents of the remaining five fetuses, including three healthy and two unhealthy fetuses, chose to terminate their pregnancies.

**Conclusion:**

The fetus and neonate phenotype of prenatally detected isochromosome Y usually is that of a normally developed male, ascertained in the absence of other indicators of a fetal structural anomaly. Our study provides clinical reference materials for risk assessment and permits better prenatally counseling and preparation of parents facing the birth of isochromosome Y fetuses.

## Background

An isochromosome is a chromosome for which the two arms are symmetrical in terms of gene type, number and arrangement. Idic(Y) is a mirror-imaged chromosome with an axis of symmetry between two centromeres, whose two mirror-imaged arms are identical and symmetrical in terms of the type, number and arrangement of genes [1, 2]. I(Y) carries one centromere and a duplication of the short or long arm. Idic(Y)/i(Y) often exists as mosaicism and has a 45,X cell line [3]. The formation of mosaicism is extremely complex and depends on the meiotic and/or postzygotic mitotic stability of cells [4, 5]. There are many reports on idic(Y) at http://cs-tl.de/DB/CA/sSMC/32XY/a-Start.html, including 141 phenotypic male cases and 52 phenotypic female cases. The mosaic patients with idic(Y)/i(Y) are associated with a wide range of phenotypic manifestations, such as Turner syndrome in females [6, 7], ambiguous genitalia [6, 8], gonadal dysgenesis [9], phenotypically normal males with infertility [6, 10], short stature [11, 12], craniofacial abnormalities [13], and male mental retardation, in postnatally ascertained cases [14]. However, in the vast majority of reported postnatally ascertained mosaic idic(Y)/i(Y) patients, karyotyping was performed due to abnormal phenotypes, obviously biasing the available data.

Here, we identified nine prenatally ascertained cases with idic(Y)/i(Y) through cytogenetic analysis, including the prenatal diagnosis and pregnancy outcomes. The abnormal Y chromosome was identified by different approaches, such as chromosomal microarray analysis (CMA), quantitative fluorescent polymerase chain reaction (QF-PCR), and fluorescence in situ hybridization (FISH), the findings of which emphasized the importance of combining conventional cytogenetic analyses with molecular techniques in prenatal diagnosis. Furthermore, we analyzed the relationship between genotypes and phenotypes. The study provides clinical reference materials for idic(Y)/i(Y) carriers' genetic counseling in prenatal diagnosis.

## Subjects

Nine gravidas who received invasive prenatal diagnosis for various indications were gathered in the Prenatal Diagnostic Center of Guangdong Women and Children Hospital from January 2015 to September 2019. All gravidas signed informed consent forms, and their clinical data, including sex, age, history of gestation, family medical history, chemical and radiation exposure history, and clinical and laboratory test results, were recorded. The study was approved by the Medical Ethics Committee of Guangdong Women and Children Hospital.

## Methods

### Cytogenetic analysis

Chromosomal specimens in amniotic fluid (AF) cells were harvested in situ, and chromosomal specimens in cord blood (CB) lymphocytes were harvested according to standard protocols. Conventional karyotyping was performed in accordance with the International System for Human Cytogenomics Nomenclature 2020 (ISCN2020) [15].

### FISH analysis

FISH analysis was carried out by AneuVysion Multicolor DNA Probe Kit (Abbott Molecular Inc., USA) following the manufacturer’s instructions. The probe was specific for the centromeric region of the chromosome.

### CMA analysis

CMA analysis was performed through the use of the Affymetrix CytoScan 750K gene chip (Affymetrix, CA, USA). Specimen preparation included genomic DNA extraction, digestion, ligation, PCR setup, PCR purification, quantitation, fragmentation, labeling, hybridization, washing, staining and scanning. Chromosome Analysis Suite software (Affymetrix, CA, USA) was applied for data analysis. The distance between the centromeres of idic(Y)/i(Y) was estimated by multiplying by two the distance separating the breakpoint and the centromere, based on the localization of breakpoints in the Genome Browser.

### QF-PCR analysis

The primers were synthesized and labeled by Takara Biotechnology (Dalian) Co., Ltd. The short tandem repeat (STR) locus of trisomy analysis in the Y and X chromosomes included AMEL, DX981, DYS448, SRY, DXYS218, DXYS267, DXS1187, DXS7423, TAF9, SY86, SY84, SY127, SY134, SY255, ZFX/Y, SY254, SY152 and SY145. The STR loci for microdeletions in the Y chromosome were SY84 and SY86 (in AZFa), SY127 and SY134 (in AZFb), SY254 and SY255 (in AZFc), SY152 and SY145 (in AZFd), and ZFX/Y and SRY gene. After PCR amplification, the products were detected by capillary electrophoresis. GeneMarker V2.2.0 software was used to analyze the peak figure data.

### Follow-up

The follow-up information included pregnancy outcomes, delivery modes, premature birth or at-term birth, and postnatal growth and development.

## Results

### Clinical materials

Seven idic(Y) fetuses and two i(Y) fetuses were identified. The nine gravidas were 25–41 years old, with an average age of 28 years old. One gravida had advanced maternal age (41 years old). The family medical history of the nine gravidas was uneventful. Fetus 2 and fetus 3 were monochorionic-diamniotic twins whose cases had been reported by Liu et al [16].

### Conventional karyotyping

Seven idic(Y) fetuses and two i(Y) fetuses were identified. Fetus 8 was complete for i(Y)(p10), and the rest with 45,X had mosaic forms. Of the fetuses with idic(Y), a break and fusion locus was found in fetus 1 in Yp11.3; fetuses 2, 3, 4, 6, and 7 in Yq11.221; and fetus 5 in Yq11.222. Fetus 8 was complete for the i(Y) form, and fetus 9, with mosaicism, carried i(Y), 45,X, 46,X,del(Y) and normal 46,XY cell lines. For four fetuses (1, 2, 5, 6), AF and CB cells were collected for karyotyping. The karyotypes of fetus 1 and fetus 2 in AF and CB cells were consistent, but the proportions of cell lines were different. A few CB lymphocytes with double idic(Y) were found in fetus 5 and fetus 6. The karyotype information of the nine fetuses is listed in Table 1.

**Table 1:** Clinical prenatal diagnosis informations and pregnancy outcomes of nine cases

Cases	Indication	Tissue	Karyotype	CMA	FISH	QF-PCR	Ultrasound findings	Clinical outcome
1	a. Was referred to our hospital due to karyotype mos 45,X[45]/46,X,+mar[8] in AF cells.	AF	mos 45,X[23]/46,X,idic(Y)(p11.3)[6]	arr(1-22)×2, (X)×1, (Y)×0-1(copies 0.75)	nuc ish (DXZl×1,DYZ3×0)[39/100]/(DXZl,DYZ3)×1[42/100]/(DXZl×1,DYZ3×2)[19/100]	X/XY mosaicism, SRY(+), AZF no deletion	N	TOP
		CB	mos 46,X,idic(Y)(p11.3)[108]/45,X[103]		nuc ish (DXZl×1,DYZ3×0)[53/100]/(DXZl,DYZ3)×1[40/100]/(DXZl×1,DYZ3×2)[7/100]			
*2	a. The twins' imbalanced development. b. Twin 2 was found fetal cleft lip, cleft palate, ventricular septal defect and fetal bilateral radius hypoplasia or absence.	AF	mos 45,X[27]/46,X,idic(Y)(q11.22)[14]	arr[hg18]Yq11.221q11.23(17,073,540-27,176,992)×0	nuc ish (DXZl×1,DYZ3×0)/(DXZl×1,DYZ3×2)	XYY or structurally abnormal Y chromosome, SRY(+)	N	TOP: Male, weight 1.1 kg, body length 37 cm, head circumference 26 cm
		CB	mos 46,X,idic(Y)(q11.22)[95]/45,X[5]		nuc ish (DXZl×1,DYZ3×0)/(DXZl×1,DYZ3×2)			
*3		CB	mos 46,X,idic(Y)(q11.22)[90]/45,X[10]		nuc ish (DXZl×1,DYZ3×0)/(DXZl×1,DYZ3×2)		Fetal cleft lip, cleft palate, ventricular septal defect and fetal bilateral radius hypoplasia or absence	TOP: Male, weight 0.6kg, body length 31cm, head circumference 21cm, cleft lip and palate, abnormal postures of hands
4	a. Fetal left ventricular bright spot. b. Excess sex chromosomes by NIPT.	CB	mos 46,X,idic(Y)(q11.22)[97]/45,X[3]	arr[hg19] (X)×1, Yp11.31q11.221(2,650,424-15,715,099)x2	nuc ish (DXZl×1,DYZ3×0)/(DXZl×1,DYZ3×2)	XYY or structurally abnormal Y chromosome, SRY(+), AZFb+c+d deletion	Fetal left ventricular bright spot	Birth: A 3.5 kg baby boy, normal development, natural birth
5	a. Was referred to our hospital due to karyotype mos 45, X/46, XY in AF cells. b. AMA.	AF	mos 45,X[19]/46,X,idic(Y)(q11.22)[3]/46,X,del(Y)(q11.22)[2]/46,XY[1]	arr[hg19] (X)×1, Yp11.31q11.222(2,650,424-20,609,790)x1-2, Yq11.222q11.23(21,035,823-28,799,654)x0	nuc ish (DXZl×1,DYZ3×0)[36/100]/(DXZl,DYZ3)×1[34/100]/(DXZl×1,DYZ3×2)[14/100]/(DXZl×1,DYZ3×4)[16/100]	XYY/XY mosaicism, SRY(+), AZFb+c+d deletion	N	Birth: A 3.25 kg baby boy, normal development, cesarean section
		CB	mos 46,X,idic(Y)(q11.22)[34] /45,X[12]/47,X,idic(Y)(q11.22)x2[5]/46,X,del(Y)(q11.22)[5]/46,XY[44]		nuc ish (DXZl×1,DYZ3×0)[25/100]/(DXZl,DYZ3)×1[31/100]/(DXZl×1,DYZ3×2)[34/100]/(DXZl×1,DYZ3×4)[10/100]	XYY/XY mosaicism		
6	a. Increased fetal NT thickness (3.6 mm).	AF	mos 45,X[14]/46,X,idic(Y)(q11.22)[3]	arr[hg19] Yq11.221q11.23(19,571,466-28,799,654)x0	nuc ish (DXZl×1,DYZ3×0)/(DXZl×1,DYZ3×2)	XY, SRY(+), AZFb+c+d deletion	Increased fetal NT thickness (3.6mm)	TOP
		CB	mos 46,X,idic(Y)(q11.22)[76]/45,X[21]/47,X,idic(Y)(q11.22)×2[3]	arr[hg19] Yp11.31q11.222(2,650,424-19,955,778)x4, Yq11.221q11.23(19,571,466-28,799,654)x0	nuc ish (DXZl×1,DYZ3×0)/(DXZl×1,DYZ3×2)			
7	a. A critical risk of trisomy 21 by early Down's screening. b. Low number of sex chromosomes by NIPT.	AF	mos 45,X[35]/46,X,del(Y)(q11.22)[5]/46,X,idic(Y)(q11.22)[3]	arr[hg19] Yq11.221q11.23(19,747,270-28,799,654)x0	nuc ish (DXZl×1,DYZ3×0)/(DXZl,DYZ3)×1/(DXZl×1,DYZ3×2)	XY, SRY(+), AZFb+c+d deletion	N	Birth: A 2.9 kg baby boy, normal development, cesarean section
8	a. Increased fetal NT thickness (3.0 mm). b. A high risk of trisomy 21 (1/140) by Down's screening. c. Sex chromosome abnormality by NIPT.	AF	46,X,i(Y)(p10)	arr[hg18] Yp11.31p11.2(2,716,461-10,379,571)×2, Yq11.21q11.23(12,571,053-27,176,992)×0	nuc ish (DXZl,DYZ3)×1	XYY or structurally abnormal Y chromosome, SRY(+), AZFa+b+c+d deletion,	Increased fetal NT thickness(3.0mm)	Birth: A 3.125 kg baby boy, normal development, natural birth
9	a. Double renal pelvis images of left kidney and permanent left superior vena cava.	AF	mos 45,X[46]/46,X,i(Y)(p10)[9]/46,X,del(Y)(q11)[5]/46,XY[5]	arr[hg19] Yq11.223q11.23(24,073,794-28,799,654)x0	nuc ish (DXZl×1,DYZ3×0)/(DXZl,DYZ3)×1	XY, SRY(+), AZFc+d deletion	Fetal double renal pelvis of left kidney and permanent left superior vena cava	TOP

AF, amniotic fluid; CB, cord blood; N, normal; TOP, termination of pregnancy; NIPT, non-invasive prenatal test; AMA, advanced maternal age; NT, Nuchal translucency.

*Fetus 2 and fetus 3 were monochorionic-diamniotic twins

### FISH analysis

The FISH test results showed that idic(Y) had two DYZ3 (red) signals, and idic(Y)x2 had four DYZ3 (red) signals. Del(Y), Y and i(Y) had one DYZ3 (red) signal. The X chromosome had one DXZ1 (green) signal. The FISH test information of the nine fetuses is listed in Table 1.

### CMA analysis

There were duplications in four fetuses (4, 5, 6, 8) and deletions in seven fetuses (1, 2, 5-9). Fetus 1 had a mosaic deletion throughout the Y chromosome (copy number 0.75). Fetus 2 had an approximately 10.1 Mb deletion on Yq11.221-q11.23. The mother of fetus 3 refused the CMA medical examination due to fetal malformations identified by B ultrasound. Fetus 4 had an approximately 13.1 Mb duplication on Yp11.31-q11.221. Fetus 5 had an approximately 18.0 Mb mosaic duplication on Yp11.31-q11.222 (copy number 1.6) and an approximately 7.8 Mb deletion on Yq11.222-q11.23. Fetus 6 had an approximately 9.2 Mb deletion on Yq11.221-q11.23 in AF cells but had an approximately 17.3 Mb tetrasomy on Yp11.31-q11.222 and an approximately 9.2 Mb deletion on Yq11.221-q11.23 in CB lymphocytes. Fetus 7 had an approximately 9.1 Mb deletion on Yq11.221-q11.23. Fetus 8 with 46,X,i(Y)(p10) had an approximately 10.4 Mb duplication on the whole Yp arm and an approximately 14.6 Mb deletion on the whole Yq arm (the heterochromatin region on Yq was not included). Fetus 9 had an approximately 4.7 Mb deletion on Yq11.223-q11.23. The CMA test information of the fetuses is listed in Table 1.

The intercentromeric distance of idic(Y) varied from 6 to 25 Mb. The breakpoints of three fetuses were involved in/near palindromes or inverted repeats (fetus 2 near P5, fetus 4 near P7, and fetus 5 near IR2) based on the localization of breakpoints in the Genome Browser.

### QF-PCR analysis

For two fetuses (1, 5), X/XY and XYY/XY mosaicism was suggested; for three fetuses (2, 4, 8), XYY mosaicism was suggested; and for three fetuses (6, 7, 9), no sex chromosome mosaicism was suggested, but the possibility of a sex chromosome structural abnormality was not excluded. The SRY gene was detected in eight fetuses (i.e., except for fetus 3), and all eight fetuses were SRY (+). A deletion in the AZF region was detected in seven fetuses (i.e., except for fetus 2 and fetus 3). The AZF region in fetus 1 was intact. Fetuses 4-7 had an AZFb+c+d deletion, fetus 8 had an AZFa+b+c+d deletion, and fetus 9 had an AZFc+d deletion. The QF-PCR test information of the fetuses is listed in Table 1.

### Follow-up

Finally, four healthy male neonates (4, 5, 7, 8) were delivered successfully, but the parents of the remaining five fetuses chose to terminate pregnancy after genetic counseling. The aborted fetuses included three healthy fetuses (1, 2, 6) and two unhealthy fetuses (3, 9). Although fetus 2 and fetus 3 were monochorionic-diamniotic twins, a cleft lip, cleft palate, ventricular septal defect and radius hypoplasia or absence were detected by B ultrasound in fetus 3, and fetus 2 was healthy. A double renal pelvis of the left kidney and permanent left superior vena cava were detected by B ultrasound in fetus 9. Detailed information on the nine fetuses is listed in Table 1.

## Discussion

### Formation mechanism of idic(Y)

**Fig. 1:**
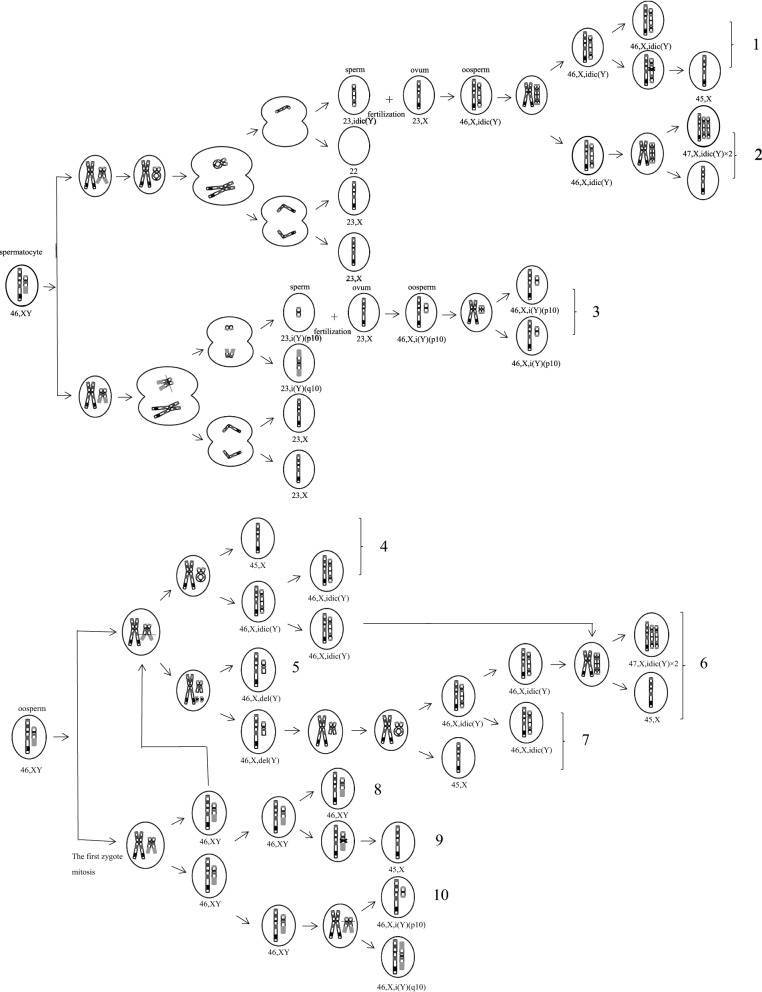
Schematic diagram of suggested mechanisms of origin of abnormal chromosomes of fetuses. *Note*: (1) or (4): mos 45,X/46,X,idic(Y) (fetus 2, 3, 4, 6 in AF cells). (3): 46,X,i(Y)(p10) (fetus 8 in AF cells). (1+2) or (4+6): mos 45,X/47,X,idic(Y)×2/46,X,idic(Y) (fetus 6 in cultured CB lymphocytes). (8+5+7): mos 45,X/46,X,idic(Y)/46,X,del(Y)/46,XY (fetus 5 in AF cells). (8+5+6+7): mos 45,X/46,X,idic(Y)/46,X,del(Y)/46,XY/47,X,idic(Y)×2. (Fetus 5 in cultured CB lymphocytes). (5+8+9+10): mos 45,X/46,X,i(Y)(p10)/46,X,del(Y)(q11)/46,XY (fetus 9 in AF cells).

The formation of idic(Y) has been attributed to breakage-fusion-bridge (BFB) cycles between sister chromatids [5]. The mechanism is as follows: the sister chromatids of the Y chromosome break in a symmetrical site, then the fusion of the fracture ends in mitosis or meiosis I, and then idic(Y) is formed [2, 17]. The remaining acentric fragment is very unstable and mostly lost during anaphase unless the spontaneous formation of a neocentromere occurs in the same cell cycle [18]. The breaks in the sister chromatids of the Y chromosome occur at different stages or in different cell lines, and fetal chromosomes may be complete or have various mosaic forms. Dynamic mosaicism usually makes mosaic karyotypes very variable [19]. The possible mechanisms of idic(Y)/i(Y) formation in fetuses are shown in Fig. 1.

### The intercentromeric distance and stability of idic(Y) and i(Y)

The origin of mosaic cell lines has been attributed to the division instability of idic(Y) due to the existence of double centromeres [3, 20]. In humans, a positive correlation between intercentromeric distance and mitotic instability has been demonstrated in cultured cells [21, 22]. Patients with an intercentromeric distance greater than 20 Mb on idic(Y) would have an increased risk of a female phenotype due to extensive 45,X gonadal mosaicism [2, 23]. To counter the instability of idic(Y), one centromere is usually inactivated, and the other constricted active centromere continues to work. The longer the intercentromeric distance is, the more heavily idicYp relies on the functional inactivation of one centromere to maintain mitotic stability [2]. However, if the intercentromeric distances are short enough, mitotic stability may remain via two centromeres functioning as one, or close proximity physically constrains them from attaching to opposite spindle poles in mitotic division [17, 21, 24]. T Haaf found that the lymphoblastoid cell lines with 46,X,i(Y) were still stable in mitosis after passage for 100 generations [25]. This means that functionally monocentric i(Y) may be mitotically stable. However, complete i(Y) prenatal reports are extremely rare, and complete i(Y) might go undetected because most fetuses have no abnormal phenotypes. In our study, an invasive prenatal diagnostic procedure was performed in fetus 8 due to increased fetal NT thickness (3.0 mm), a high risk of trisomy 21 (1/140) in Down's syndrome screening, and sex chromosome abnormality identified by NIPT. Finally, the fetus was diagnosed as being complete i(Y)(p10), and a 3.125 kg boy was naturally born with normal development.

### The susceptibility to the formation of idic(Y)

The human ampliconic region contains 8 large palindromes made up of inverted repeats that span 5.7 Mb located at Yq11.22-Yq11.23, and there are also three other long inverted repeats (IR1-3) in the ampliconic male-specific region of the Y chromosome (MSY) [26]. Lange et al. found that 56 of 78 patients with idic(Y) had breakpoints in palindromes, and all inverted repeats on Yq (P1-P6, P8 and IR2) but P7 were involved [2]. Bergeron et al. found that the breakpoints of 4 of 10 patients with idic(Y) were in/near palindromes or inverted repeats [23]. In our study, the breakpoints of three fetuses were involved in/near palindromes or inverted repeats, consistent with previous reports. The findings from these studies suggested that the occurrence of exact crossover recombination pathways (unequal sister exchanges) is active in the palindromic regions of the Y chromosome, albeit at a very low frequency [2, 27]. However, common fragile sites of chromatin, such as AT-rich sequences, are prone to breakage and fusion [28, 29]. Therefore, although palindromes and repeat sequences might confer susceptibility to the formation of idic(Y), these sequences were not implicated in all patients.

### The deletion/duplication effect and phenotypes

Although idic(Y)/i(Y) with an extra *SRY* copy has been reported in well-known phenotypes, it were not considered in this instance, as this usually occurs in the background of other genes involved in structural rearrangement or whole chromosome gain. There was no substantial evidence for focal duplications involving only *SRY* leading to corresponding clinical phenotypes [30]. In fetuses with a double *SRY* copy, the fetus usually develops as a male with normal external genitalia, as in the case of 47,XYY syndrome [31, 32]. In our study, 7 of 9 fetuses with two copies of *SRY* showed no abnormal phenotypes.

Of more importance seems to be the extent of deleted genetic information on the derived Y chromosome. The AZF region containing the genes controlling spermatogenesis is located in Yq11.2, which encompasses the AZFa, AZFb and AZFc regions [33]. The entire deletion of the AZFa region results in Sertoli cell only syndrome (SCOS) and azoospermia [34–40]. The deletion of the AZFb region results in the testicular phenotype of maturation arrest [41, 42]. Men with AZFc deletion have the most variable phenotype, ranging from complete azoospermia to mild oligozoospermia [43]. AZFd deletion is generally considered to be a polymorphic variation. The AZFb+c+d deletion of fetuses 4-7 will lead to hypospermatogenesis or azoospermia in adulthood. Complete fetus 8 with i(Y) completely deleted in the total AZF region and will show azoospermia in adulthood. Although the idic(Y)(p11.3) of fetus 1 had a double AZF region, fetus 1 carried the 45,X cell line. Therefore, the level and distribution of the 45,X cell line in gonads might affect spermatogenesis.

### Mosaic levels and clinical phenotypes

Bergeron et al. found that the proportion of idic(Y) cell lines can vary greatly among tissues and was generally higher in gonads than in blood [23]. Jie Xu et al. found that > 20% of G-banded amniocytes with idic Yp seemed to correlate with phenotypically healthy males in most cases in the absence of other indicators of fetal structural anomalies [44]. High levels of the mosaicism of 45,X and low levels of idic(Y)/i(Y) cell lines in gonads may lead to mosaic loss, the haploinsufficiency of *SRY*, and an inability to maintain normal testosterone differentiation, thus manifesting in ambiguous genitalia or a female phenotype [45–48]. However, a lack of correlation between the level of mosaicism in AF cells and the phenotypic sex has been reported [49–51]. In our study, except for complete fetus 8, the proportion of idic(Y)/i(Y) cell lines varied from 7 to 34% in AF cells and 39–97% in CB lymphocytes, and the proportion of 45,X cell lines was from 66 to 82% in AF cells and 3–49% in CB lymphocytes. Although fetus 2 and fetus 3 were monochorionic-diamniotic twins with similar mosaic levels in CB lymphocytes, fetus 2 was a healthy male fetus, and fetus 3 had multiple structural malformations as identified by ultrasound. Fetal structural abnormalities were not associated with the mosaic level in our study. However, our sample size was very small, and the statistical results might be biased.

### Limitation of AF and CB specimens

The cells in AF, which are a mixture of exfoliated cells from multiple germ layers, come from a variety of sources [52]. Lymphocytes in the CB originate from the lateral plate mesoderm [53]. Most organs and structures of the genitourinary system originate from the intermediate mesoderm [54]. However, the mosaic level in gonads cannot always be accurately assessed due to the inability to obtain cells from gonadal tissue in prenatal diagnosis. The limitations of prenatal specimen sources and cell culture often lead to inconsistent and biased results of the proportion and types of cell lines in prenatal diagnosis. It is difficult to evaluate the severity of phenotypes in gonads because the origin of the germ layer and the true mosaic level of tissues and organs cannot be distinguished.

### The combined application of multiple technologies

Abnormal cell division and growth in in vitro culture may lead to the inherent bias of dominant clone culture in karyotype preparation. Therefore, the traditional concept of karyotyping as the "gold standard" should be amended appropriately in the diagnosis of idic(Y)/i(Y). The uncultured specimens can be analyzed by FISH, CMA and QF-PCR, which can better reflect the real copy number. FISH can identify not only the number and structural abnormalities of sex chromosomes but also low level mosaicism, i.e. less than 10% [55]. And vice versa, CMA can detect chromosome mosaicism only as low as 10% [56]. QF-PCR can detect the copies of the SRY gene and AZF region [57]. The complementarity of multiple techniques can better determine the composition and proportion of mosaicism.

### Clinical prognosis and prenatal genetic counseling

With the rapid development of molecular genetic technology, an increasing number of idic(Y)/i(Y) fetuses are detected prenatally. The prevalence of noninvasive prenatal testing (NIPT) is one of the main reasons for the increased detection rate of sex chromosome abnormalities. In our study, normal phenotypes of fetuses/neonates were reported in 7 out of 9 cases, in contrast with previously reported postnatally ascertained idic(Y)/i(Y). These results suggest that the phenotype of fetuses/neonates in whom idic(Y)/i(Y) is prenatally identified is usually that of a male with normal development, ascertained in the absence of indicators of a fetal structural anomaly. There are many cases of prenatally identified normal male phenotypes, but cases of prenatally identified normal female phenotypes with idic(Y)/i(Y) are extremely rare [23, 44, 49, 51, 58–61].

The prenatal consultation for such patients can be summarized with the following 5 points: A. The proportion of cell lines may not be clearly related to phenotypes. Karyotyping is not the “gold standard” because of the bias in cell culture. The mosaic level identified in FISH, CMA and QF-PCR tests is more reliable due to the uncultured cell specimens. However, these tests cannot identify the mosaic level of gonads. AF cells represent the overall fetal level of mosaicism, and CB lymphocytes represent the level of mosaicism in organs and tissues derived from the mesoderm. B. The phenotype of the fetus/neonate is usually a normal developed male, ascertained in the absence of other indicators of a fetal structural anomaly. C. Patients do not have normal fertility upon maturation, which is associated with the complete or partial absence of the AZF region and mosaic levels of multiple cell lines in the gonads. D. There is an increased risk of gonadoblastoma. E. Abnormalities might arise during puberty. Clinical management will be very important, along with the follow-up of the growth and development of the affected individuals.

## Conclusion

Although most idic(Y)/i(Y) fetuses/neonates show no abnormal phenotypes prenatally, some parents still choose to terminate pregnancy after genetic counseling. Prenatal genetic counseling about idic(Y)/i(Y) certainly constitutes a challenge, yet it is believed that our study provides clinical reference materials for risk assessment and permits better prenatal counseling and preparation of parents with idic(Y)/i(Y) fetuses.

## Data Availability

The datasets used and/or analyzed during the current study are available from the corresponding author on reasonable request.

## Abbreviations

CMA: Chromosomal microarray analysis
QF-PCR: Quantitative fluorescent polymerase chain reaction
FISH: Fluorescence in situ hybridization
AF: Amniotic fluid
CB: Umbilical cord blood
ISCN2020: International System for Human Cytogenomics Nomenclature 2020
STR: Short tandem repeat
IR: Inverted repeats
MSY: Male-specific region of Y chromosome
SCOS: Sertoli cell only syndrome
NIPT: Noninvasive prenatal testing
